# The effect of integrating midwifery counseling with a spiritual content on improving the antenatal quality of life: A randomized controlled trials

**DOI:** 10.34763/jmotherandchild.20222601.d-22-00003

**Published:** 2022-11-02

**Authors:** Masoumeh MonfaredKashki, Azam Maleki, Kourosh Amini

**Affiliations:** Student in Counseling in Midwifery, School of Nursing and Midwifery, Zanjan University of Medical Sciences, Zanjan, Iran; Social Determinants of Health Research Center, Zanjan University of Medical Sciences, Zanjan, Iran; Department of Mental Health Nursing, School of Nursing and Midwifery, Zanjan University of Medical Sciences, Zanjan, Iran

**Keywords:** Quality of life, Pregnancy, Spirituality, Women’s Health, Childbirth

## Abstract

**Background:**

Poor antenatal Quality of Life (QoL) is associated with adverse outcomes.

**Objective:**

This study was performed to examine the effect of integrating midwifery counseling with spiritual content on improving the antenatal quality of life.

**Method:**

This randomized controlled trial was performed on 60 first-time pregnant women who were referred to two childbirth preparation centers in Zanjan city, Iran in 2019. The counseling was conducted in eight sessions. The QoL SF-36 questionnaire was completed before and two months after the intervention. Data were analyzed using the chi-square test, independent t-test, and paired-samples t-test. The level of significance was p<0.05.

**Results:**

After intervention based on an independent t-test the total score of QoL was significantly greater in the intervention group compared to the control group (p=0.001). After the intervention, the mean scores of four domains of QoL (Role-Physical, General Health, Vitality, Role-Emotional, and Mental Health) were significantly higher than the control group(p=0.001). While in terms of Physical Functioning, Bodily Pain and Social Functioning domains were not statistically significant (p>0.05).

**Conclusion:**

Integrating midwifery counseling with spiritual content had a positive impact on improving the psychological aspect of quality of life more than the physical and social aspects. It can be used by providers for planning antenatal care programs.

## Introduction

Pregnancy is a physiological phenomenon in a women’s life. Physical and psychological changes during pregnancy can affect the social and physical performance, as well as the quality of life (QoL) of pregnant women [1]. The quality of life (QoL) reflects the subjective perceptions of the individual’s situation in life-based on the cultural and value system, given the individual’s goals, expectations, standards, and attitudes [2]. According to the World Health Organization (WHO), health-related QoL refers to the physical, psychological, social, and spiritual dimensions of individuals’ well-being [3]. Furthermore, the QoL of pregnant women could be affected by many factors such as gestational age, social and economic support, and complications before or during pregnancy [4]. On the other hand, poor pregnancy QoL is associated with adverse outcomes for example preterm labor pain, and pregnancy-related symptoms such as fatigue, and low back and pelvic pain[5]. Additionally, low QoL in pregnancy contributes to low QoL in the postnatal period [6].

Spirituality is known as an important component of health and well-being. Although the concepts of religion and spirituality are similar in some aspects and are often used interchangeably, they mean different meanings. Spirituality is a way of perceiving the sublime, understanding certain values and goals of life, and experiencing positive and satisfying behaviors and emotions in life through non-physical methods [7]. In this respect, spiritual care can be educated in nursing and midwifery to be able to provide spiritual care as part of holistic and person-centred care [8, 9].

Childbearing is one of the ideal conditions for enriching spirituality. Some people believe that the process of pregnancy and childbirth is a time to get closer to God and make life more meaningful [10]. Spirituality is defined as sensitivity or attachment to religious values, or to things of the spirit as opposed to material or worldly interests. Spiritual experience is a unique experience and includes understanding the meaning of life, positive life experiences, feeling happy, and life satisfaction [11].

In Iran, spiritual care has not been routinely included in antenatal care programs, while in recent years, valuable results from the implementation of interventions based on religion and spirituality in improving anxiety, depression, and coping with stress have been reported [12]. The use of spiritual counseling alone or in combination with cognitive-behavior therapy can improve QoL in women with a high-risk pregnancy, postpartum depression, and fear of labor pain [13, 14, 15]. However, there is a gap in the effectiveness of spiritual-based interventions in the culture and context of Iran on health-related QoL in women with the first pregnancy. Given the importance of spiritual care and the presence of limited studies in this field, this study aimed to examine the effect of integrating midwifery counseling with spiritual content on improving the antenatal quality of life.

## Material and methods

### Study aim and design

This parallel randomized controlled trial was performed to examine the effect of integrating midwifery counseling with spiritual content on improving the antenatal quality of life among first-time pregnant women.

### Setting

The study was performed on 60 first-time pregnant women who were referred to two childbirth preparation centers in Zanjan a city in northwest Iran, in 2019. There are three childbirth preparation centers in Zanajn. One of the childbirth preparations centers is located in a hospital and covers most high-risk pregnancies, so sampling was done from only two centers that provide services in the urban health community center.

### Participants

The study population included first-time pregnant women who were referred to two childbirth preparation centers in Zanjan. Inclusion criteria consisted of living in Zanjan city, gestational age of 20-24 weeks, willingness to participate in the study, obtaining scores ≤10 according to the Edinburgh Postnatal Depression Scale (EPDS) [16], scores of 19 to 37 based on the Cohen Perceived Stress Scale (PSS-14) [17] and having a normal pregnancy with a singleton fetus.

Exclusion criteria before randomization were the presence of medical or obstetric complications, psychiatric disorders or use of psychiatric drugs, and no access to telephone for follow-up. There was no attrition in the study and after the interventions.

### Procedure

**Figure 1:**
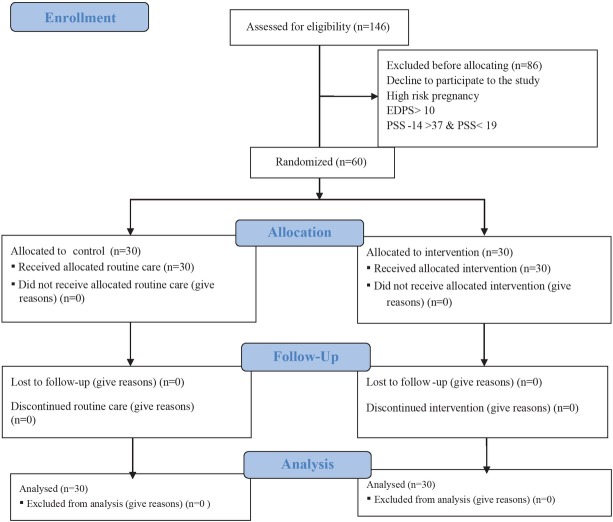
Flow chart of the participant’s selection.

Pregnant women who met the inclusion criteria and signed the informed consent form were allocated into two intervention and control groups using randomized a block size of four. To ensure the concealment of the sequence of enrolment, an opaque sealed envelope system was used [18]. Envelope preparation and random allocation sequencing were performed by a person not involved in the research process. In the present study, participants & researcher were not blinded only outcome assessors were blinded. The research process is shown in Figure 1.

### Intervention

The counselling was conducted by a midwife (the first author) familiar with counselling approaches. The content of the sessions was developed under the supervision of a spiritual advisor by following the study of khodaKarmari et al.[19] and the method suggested by Richard and Bergin [20]. The intervention group received eight sessions of counseling in addition to routine care. The counseling was held in 8 sessions, as a group counseling (8-10 people) for 4 weeks (2 times per week for 45 minutes) in preparation classrooms. The counseling was conducted by a midwife (the first author) who that familiar with counseling approaches under the supervision of a clinical psychologist. Educational content was prepared using the Holy Quran and religious books (Hadis) and integrated with routine midwifery counseling. The main topic of counseling was reported in Table 1.

**Table 1:** Details of the intervention

Sessions	Counseling content	Session homework
**First**	The first session was to meet the participants and researcher, to explain the aim, the rules, and the brief full program. Providing pre-test. Talking about the concept of quality of life, self-concept in pregnancy, and checking misconceptions. Focus on human creation and discussing concerning the status of women in the continuity of creation	Practice looking at their life issues from other angles. Prepare a list of pregnancy stressors. Be aware of the stresses that accumulate all over your body and make you suffer.
**Second**	Assessing attitudes and beliefs of the pregnant woman on spiritual issues, the role of god, and religion in her life. Talking about the spiritual aspects of pregnancy and childbearing. Listening to the physical and mental problems, worries, fears, ambivalence sense in early pregnancy and her actions in daily life.	Visualize unfavorable conditions and try to switch appropriate and positive reactions in your mind to inappropriate ones.
**Third**	Discussing the positive effects of helping each other in our life. Finding the truth of their existence, not just addressing personal desires in pregnancy Focus on the concept of trust, resort, patience, kindness. Listening to positive statements of participants based on reading the holy book, and spiritual issues in overcoming or feeling calm in stressful situations.	Create a daily spiritual space of time or place at home. Reflect on what others are saying and pay attention to the root cause of others’ behavior or speech and try not to react too quickly.
**Fourth**	Blessings of God and the role of it in reinterpreting the concept of pregnancy and overcoming the worrisome symptoms of pregnancy. Strengthening individuals’ inner hope and powers for coping with pregnancy and childbearing. Teaching relaxing muscles with deep breathing for getting rid of the stress. Repeat twice daily for 10 to 15 minutes	Book therapy / listening to Qur’an voices for 10 min.
**Fifth**	Discussing the role of patience and trust in God in enduring the pain of childbirth and the spiritual reward of pregnancy, childbirth, and breastfeeding for the mother. Encouraging to express their feeling after/ during creating a daily spiritual space. Talking about the experience of participating in religious programs or doing spiritual issues. Discuss the effect of spiritual’s beliefs on eating habits on the fetus, taking care of oneself in pregnancy.	Listening to “Nature’s Music” the sound of birds, rivers, and waterfalls…
**Sixth**	Encouraging to refer to people who create a positive atmosphere or with which she feels comfortable. Illustration and slowly moving tone using meditation relaxation technique along or with listening to relaxing music Take a realistic look at the issues and changes that have taken place in their pregnancies Do not be strict or easy-going. Allocate as much attention and time as needed to each issue	Book therapy / listening to Qur’an voices for 10 min.
**Seventh**	Pay attention to the concepts of resentment, unforgiveness, guilt, and forgiving oneself and others. Discuss the strategy of prayer therapy to reduce some symptoms of pregnancy related to pregnancy and increase hope Express the pleasure and responsibility of being a mother from the point of view of the Quran” Divine Responsibility Reward”	Focus on motherhood and look at pregnancy in terms of your productive and fertile period Enjoy the hardships of pregnancy and childbirth happily. Face these hardships and endure it
**Eighth**	Reviewing and summarizing the previous sessions’ topics	Relaxing muscles with deep breathing for getting rid of the stress. Repeat twice daily for 10 to 15 minutes

Each session was started with a focus on breathing exercises or the sacred name like “Allah”. Next, the counselor described the subject of the meeting and encouraged the mothers to express emotions, needs, concerns, and thoughts on pregnancy. At the same time, the counselor guided the participants to increase their knowledge to choose the appropriate remedy for emotional reactions during pregnancy and pay attention to spiritual aspects of life. Further advice was given as homework. At the end of each session, explanations and summaries were provided and the women discussed the topic.

According to the guidelines of the Iranian Ministry of Health, routine childbirth preparation classes were held from the 20th week of gestation every two weeks until the 32nd week of gestation. The sessions focused on making the mothers familiar with the different stages of pregnancy from fertilization to delivery, personal hygiene, nutrition, mental and physical changes during pregnancy, pregnancy risks, childbirth planning, postpartum health, breastfeeding, and child care. However, no spiritual content was included. The control group only received routine care.

### Outcomes

The main outcome of this study was to determine antenatal QoL of first-time pregnant women which were collected using the SF-36 as a standard questionnaire of QoL, which was completed by the participants before and two months after the last session.

### Data collection instruments

#### Demographic

It included personal information about a woman’s age, education, occupation, and spouse’s occupational status.

### Health-related quality of life (HRQoL) *-SF-36*

SF-36 a multidimensional measure evaluating health-related quality of life. It is widely used in clinical research and is a reliable and valid measure of health-related QoL in different populations [21, 22]. It measures the perceptions of health-related QoL in eight domains of health status: physical functioning (10 items); physical role limitations (four items); bodily pain (two items); general health perceptions (five items); energy/vitality (four items); social functioning (two items); emotional role limitations (three items) and mental health (five items). Responses are scored on a 5-point scale, that is transformed into a score of 0–100 with higher scores indicating better functioning or well-being. The validity and reliability of The Persian version of the questionnaire have been assessed by Montazeri et al. [21].

### Data analysis

The statistical analysis was performed using the SPSS software version 16. Considering the 95% confidence level (Z1-α = 1.96), the test power of 80% (Z1-β = 0.85) and based on the QOL variable in Zamani’s study with the mean and standard deviation in the intervention group (M1=32.10 and S1=2.63), control group (M2=25.90 and S2=2.33), an attrition rate of 15% the sample size of was calculated for 30 pregnant women in each group [15].

Descriptive statistics were employed to describe demographic data. The chi-square test was used to compare the demographic characteristics of the groups. The Kolmogorov-Smirnov test revealed that the scores of the QoL and its components had normal distributions. Therefore, to compare total scores and all domains between and within the groups in pre-and post-intervention, the independent t-test, and paired samples t-test were applied, respectively. The level of significance was p<0.05.

## Results

Among 146 pregnant women evaluated by the researcher, sixty women met the eligibility criteria for the study

### Demographic characteristics

The demographic data are shown in Table 2. Most of the participants were housewives and have academic-level education. There were no statistically significant differences between the two groups before the intervention in terms of demographic characteristics. The mean (SD) of gestational age in the counseling group and control group were 21.80 ± 1.27 and 21.60 ± 1.40 weeks, respectively. Also, term of the mean age of the participants and gestational age was not statistically significant between the two groups (p<0.05) (Table 2)

**Table 2:** The comparison of number (percent) of socio-demographic characteristics between two groups

Variable	Groups					P-value
	Intervention		Control Number (percent)			
	Frequency	Percentage	Frequency	Percentage		
Woman’s Education woman	Guidance High school Diploma Academic	2 1 12 15	6.7 3.3 40 50	2 1 10 17	6.7 3.3 33.3 56.7	0.44
Woman’s Employment	Employed Housewife	14 16	46.7 53.3	10 20	33.3 66.7	0.43
Spouses’ employment	Employed Unemployed	17 13	56.7 43.3	21 9	70 30	0.42
Age (years)	Mean ± standard deviation	25.80 ± 6.37		24.30 ± 6.80		0.38
Gestational age (week)		21.80 ± 1.27		21.60 ± 1.40		0.56

The mean (SD) of PSS-14 in the intervention group was 23.57 ± 3.81 and in the control group was 23.09 ± 4.29. In pre-intervention based on an independent t-test, the total score of PSS-14 was not statistically significant between the two groups (p=0.399).

The mean (SD) of the Edinburgh Postnatal Depression Score (EPDS) in intervention and control groups was 8.43 ± 1.56 and 8.40 ± 1.68, respectively. In pre-intervention based on an independent t-test, the total score of EPDS was not statistically significant between the two groups (p=0.092). All participants met the eligibility criteria for the study due to the scores of EPDS being lower than 10 and the scores of PSS-14 being between 19 to 37.

### Health-Related Quality of Life (HRQoL)

Intervention group before counseling the mean score of overall QoL was 85.66 ± 5.44 which increased to 96.46 ± 4.44 and in the control group, it was 86.86 ± 3.36 before intervention that decreased to 85.76 ± 4.04 two months after the intervention. The observed differences between the two groups were statistically significant after intervention (p=0.001).

After intervention based on an independent t-test, the mean score of four domains of QoL (Physical Role Limitations, General Health, Vitality, Role-Emotional, and Mental Health) in the counseling group was significantly higher than the control group (p=0.001). While in terms of Physical Functioning, Bodily Pain and Social Functioning domains were not statistically significant (p>0.05).

Comparing within-the group (before and after) scores of QoL and its domains in the control group showed no statistically significant differences (p>0.05).

Comparing within-the group (before and after) scores of QoL and the domains of “Physical Functioning, Physical Role Limitations, General Health, Vitality, Role-Emotional, and Mental Health” in the intervention group showed statistically significant improvements (p<0.05). Only the scores of two domains of “Bodily Pain, Social Functioning” were not statistically significant (p>0.05) (Table 3).

**Table 3:** The comparison of quality-of-life (QoLSF-36) and its domains scores between two groups

		Intervention		Control		
SF-36 Domains		Mean	SD	Mean	SD	P-value
Physical functioning	Pretest	26.75	8.43	30.25	5.14	0.05
	Post-test	31.08	8.29	27.58	5.62	0.06
P-value		Paired t-test = 0.0001		Paired t-test = 0.07		
Bodily pain	Pretest	32.91	22.62	33.75	16.78	0.87
	Post-test	33.75	25.24	38.33	20.74	0.44
P-value		Paired t-test = 0.73		Paired t-test = 0.34		
physical role limitations	Pretest	6.25	5.91	5.20	5.46	0.48
	Post-test	13.33	7.65	6.25	5.44	0.0001
P-value		Paired t-test = 0.0001		Paired t-test = 0.16		
Emotional role functioning	Pretest	16.66	8.47	16.66	8.47	1
	Post-test	29.16	9.22	16.94	8.32	0.0001
P-value		Paired t-test = 0.0001		Paired t-test = 0.91		
Social role functioning	Pretest	35.41	12.74	37.08	8.97	0.56
	Post-test	38.33	11.80	37.91	8.97	0.87
P-value		Paired t-test = 0.18		Paired t-test = 0.72		
Mental health	Pretest	57.00	7.61	58.50	6.45	0.41
	Post-test	62.33	8.78	57.16	6.78	0.01
P-value		Paired t-test = 0.0001		Paired t-test = 0.45		
Vitality	Pretest	49.79	8.44	49.16	8.64	0.77
	Post-test	63.33	4.85	49.16	8.64	0.0001
P-value		Paired t-test = 0.0001		Paired t-test = 0.18		
General health perceptions	Pretest	35.16	7.59	33.83	5.20	0.43
	Post-test	46.66	7.46	32.33	4.09	0.0001
P-value		Paired t-test = 0.0001		Paired t-test = 0.34		
Total Quality of Life Score	Pretest	85.66	5.44	86.86	3.36	0.31
	Post-test	96.46	4.44	85.76	4.04	0.0001
P-value		Paired t-test = 0.0001		Paired t-test = 0.23		

## Discussion

The study was done to examine the effect of integrating midwifery counseling with spiritual content on improving the antenatal quality of life among first-time pregnant women. Our results showed that integrating midwifery counseling with spiritual content could be improved the overall QoL. However, three domains of SF-36 QoL (physical function, bodily pain, and social function) showed no improvements. The current study emphasized that integrating midwifery counseling with spiritual content improved the psychological aspects of QoL more than the physical and social aspects. Limited information is available on the effectiveness of spiritual-based education for improving the QoL of first-time healthy pregnant women. However, our results were consistent with some studies that were conducted on multiparous or high-risk pregnancy samples. In this regard, Moazedi et al 2018, showed that Islamic teaching-based religious-spiritual psychotherapy could be improved the quality of life of infertile women [13]. The content of the counseling in our study was similar to their study and include religious and spiritual instruction in increasing the acceptance of pregnancy and responsibility through attention to the spiritual reward of pregnancy in the presence of God, the increase of trust, reflection on human creation and the greatness of creation, blessings of God and the role of it in reinterpreting the concept of pregnancy and overcoming the worrisome symptoms of pregnancy. Strengthening individuals’ inner hope and powers for coping with pregnancy and childbearing.

Zamani et al (2018) in a semi-experimental study with a pretest-posttest design showed that integrating cognitive-behavioral therapy with Islamic spirituality instructions had an effective impact on the quality of life of pregnant women [15]. Beigi et al. (2015) showed that the implementation of group spiritual therapy was effective in reducing anxiety and increasing the quality of life of women with gestational diabetes [23]. Also, similar efficacy has been reported in another study by Niaz Azari et al. in 2017 [14]. Constituency in results emphasized that the spiritual-based approach can be used to improve the quality of life of women in the antenatal and postpartum periods. According to the World Health Organization recommendation, ‘every woman has the right to the highest attainable standard of health, which includes the right to dignified, respectful health care throughout pregnancy and childbirth. The respect for pregnant women’s overall needs and their satisfaction leads to a holistic women-centered approach to care [24].

Various ideas that have been reported concerning the biological and psychological effects of spiritual experience on diseases have been emphasized in some studies. It can be claimed that some cognitive patterns, psychological characteristics, and behavioral patterns created by spirituality-oriented methods lead to strengthening health and improving the physiological function of the body and consequently increase the psychological resistance of the person in poor physical and social situations. Accordingly, spiritual practices and the religious aspect of spirituality lead to increased tolerance, patience, self-control, satisfaction, emotional control, optimism, self-efficacy (based on trust in God’s blessing), altruism, kindness, and love [25, 26]. Religion and spirituality can increase QoL by changing people’s attitudes, increasing their sense of responsibility towards themselves and others, promoting the search for meaning in life, and having a greater sense of happiness and self-esteem [27].

The effectiveness of the spiritual approach on improving QoL in the different populations [28, 29, 30] has been shown that spirituality is a universal element [31]. Belief in God creates a change in the perspective toward life [19]. The spiritual aspects of pregnancy and childbearing are often neglected in the literature. Integration of midwifery-led counseling with the spiritual approach for improving the quality of life of women is necessary. It can be concluded that spiritual counseling had a positive impact on improving the QoL of first-time pregnant women. The integration of spiritual counseling with the educational content of childbirth preparation can improve the psychological aspect of QoL of pregnant women more than the physical and social aspects. Therefore, it can be used for planning suitable interventions among pregnant women.

### Strengths of study

All the principles of control trial studies were observed in this study and we don’t have a loss of following in participants. Data collection tools were standard and psychometric properties of the Persian form of the questionnaires have been evaluated based on Iranian culture.

### Limitations

The sample size was small and the follow-up period was short. Also, samples were limited to the participants of childbirth preparation classes with moderate levels of perceived stress, which can affect the generalizability of findings. Also, the long duration of each session could be led to the exhaustion of mothers. However, the women were allowed to have rest and walk for a few minutes during the sessions.

In the present study, the spiritual intelligence of the participants was not examined before the intervention and is considered a limitation. It is suggested that additional studies should be performed by measuring spiritual intelligence and perceived stress with the long follow-up period, and participation of their spouses in future studies. Furthermore, for better conclusions about the long-term effects of the spiritual-based intervention on antenatal QoL, studies with a mixed-method design are needed to be conducted in the future.

### Key points

Poor antenatal Quality of Life (QoL) is associated with adverse outcomes.

The integration of spiritual counseling with antenatal care can improve the QoL of pregnant women.
